# Association of Maternal Anemia and Adverse Fetal Birth Outcomes Among Women Who Gave Birth at Public Hospitals in Southern Ethiopia: An Unmatched Case–Control Study

**DOI:** 10.1155/anem/9108578

**Published:** 2026-07-20

**Authors:** Serawit Lakew Chillo, Endrias Markos Woldesemayat, Mesay Hailu Dangisso

**Affiliations:** ^1^ Department of Nursing, College of Medicine and Health Sciences, Arba Minch University, Arba Minch, South Ethiopia, Ethiopia, amu.edu.et; ^2^ Department of Public Health, College of Medicine and Health Sciences, Hawassa University, Hawassa, Sidama, Ethiopia, hu.edu.et; ^3^ Ethiopian Public Health Institute, Addis Ababa, Ethiopia, ephi.gov.et

**Keywords:** adverse fetal outcome, hospital, maternal anemia, South Ethiopia

## Abstract

**Background:**

In Ethiopia, approximately 10% of births are affected by adverse fetal birth outcomes. Yet, data was limited on the relationship of anemia with adverse fetal birth outcomes in Ethiopia, particularly in the study region. This study reported the association of maternal anemia and adverse fetal birth outcomes among women who gave birth at public hospitals in southern Ethiopia.

**Methods:**

A multicenter, unmatched case–control study was conducted from May 28 to July 27, 2024, in four selected public hospitals in the study region. Data was collected using a structured and pretested questionnaire. A total of 433 participants were randomly selected, where 152 were cases and 281 were controls. The logistic regression model was fitted to detect statistical associations between the outcome and predictors at a *P* value of < 0.05.

**Results:**

The majority of participants were in the age range of 20–35 years. Types of adverse fetal birth outcomes reported range from 10 (2.3%) others (asphyxia, anomaly, and post‐term births) to 53 (12.2%) low birth weights. The overall anemic participants (anemia immediately before birth) among cases were 68 (44.7%), and controls were 48 (17.1%). The proportion of anemia status was highest with the stillbirths, 19 (73.1%), and lowest with the preterm births, 4 (20%). Participants with anemia status (immediately before birth) were 2.19 (95% CI: 1.26, 3.80) times more likely to have adverse fetal birth outcomes than non‐anemia. Participants who have better adherence to iron–folic acid supplementations during pregnancy were 52% (AOR: 0.48; 95% CI: 0.28, 0.80) less likely to face adverse fetal birth outcomes. Participants who had a low level of educational attainment, a previous history of adverse fetal birth outcomes, and intermenstrual bleeding (current history) were likely to face adverse fetal birth outcomes in the current birth.

**Conclusions:**

Anemia has an association with adverse fetal birth outcomes. Adherence to iron–folic acid in pregnancy had a protective association with adverse fetal birth outcomes. Interventions to facilitate anemia prevention strategies in pregnancy should be encouraged.

## 1. Background

Adverse fetal birth outcomes are events related to childbirth, such as a preterm birth, low birth weight, macrosomia, stillbirths, post‐term birth, neonatal asphyxia, and birth defects (such as structural and functional abnormalities) [1, 2]. Approximately, one in 10 babies was born preterm, and one in 7 newborns was low birth weight in 2020 [3, 4]. One stillbirth occurs every 17 s in 2021 worldwide [5, 6]. Countries with the highest stillbirth rates are reporting the highest prevalence of anemia in pregnancy [7, 8].

The WHO declares anemia a public health concern globally [7]. The World Bank’s 2023 report shows the highest anemia prevalence in South Asia (45%) and Sub‐Saharan Africa (43%) [9]; similarly, the prevalence of adverse fetal birth outcomes was higher in South Asia [10] and Sub‐Saharan Africa [11]. Anemia is a severe public health concern in low‐income (44%) and lower middle‐income countries (42%) [9], and a high prevalence of adverse fetal birth outcomes is reported in the region [11]. However, in the regions with decreased prevalence of adverse fetal birth outcomes [12], anemia prevalence was in the range of mild to moderate public health concern, such as in Europe and Central Asia (24%), European Union countries (18%), North America (11%), and the Western Pacific (24.3%) [9].

As justified, an adverse fetal birth outcome was related to reduced blood supply to the fetus during pregnancy by impaired maternal oxygen transport as a result of low blood hemoglobin (HGB) levels [13, 14]. Additionally, the occurrences of adverse fetal birth outcomes may be associated with a positive syphilis test in pregnancy [15], history of induced labor [15], prolonged rupture of membranes [16, 17], primiparity [18], and short interpregnancy intervals [19, 20].

In low‐ and middle‐income countries, 19% of preterm births, 12% of low birth weights, and 18% of stillbirths were attributed to anemia in pregnancy [21]. The population‐based survey reports from Ghana [22], India [23], Tanzania [24], and Bangladesh [25] had a high prevalence of adverse fetal birth outcomes and, at the same time, a high prevalence of anemia reports in 2021 and 2024 [21–25]. Anemia has an association with preterm births in late pregnancy [26, 27]. Women in a third trimester with anemia had a higher risk of low birth weight and preterm births [27, 28].

In the high‐prevalence areas of stillbirths in Ethiopia–Somalia (2018), the prevalence of anemia in pregnancy was high (in 2016) [29, 30]. Ethiopia reported an overall prevalence of low birth weight (10%), prematurity (8.76%), stillbirths (7.1%), and anomalies (2.5%) [31] between 2013 and 2019. The recent (2021 and 2024) hospital‐based studies in the Bale Zone of the Oromia Region reported 21% and, in Gondar (northern Ethiopia), 28% of all types of adverse fetal birth outcomes [32, 33]. In South Ethiopia (Wolaita Zone), a single hospital‐based study on the prevalence of stillbirths reported 8.7% [34] in 2019.

Factors associated with adverse fetal birth outcomes in Ethiopia were maternal anemia in pregnancy [35, 36], antepartum hemorrhage [33], former stillbirths [34], no SVD delivery [34], fever in pregnancy (> 2 weeks) [33], rural residence [34], teenage pregnancy (< 20 years) [34], and high‐risk pregnancy [34]. The national study (Ethiopia DHS) reports 1.17% of stillbirths and 13% of low birth weight [37] in Ethiopia. However, evidence was limited in Ethiopia to link maternal anemia in pregnancy and adverse fetal birth outcomes. Additionally, no evidence was found to link anemia in pregnancy and adverse fetal birth outcomes in the study locations of South Ethiopia. Hence, this study reports the association of anemia in pregnancy and adverse fetal birth outcomes.

## 2. Methods and Materials

### 2.1. Study Area, Period, and Design

**FIGURE 1:**
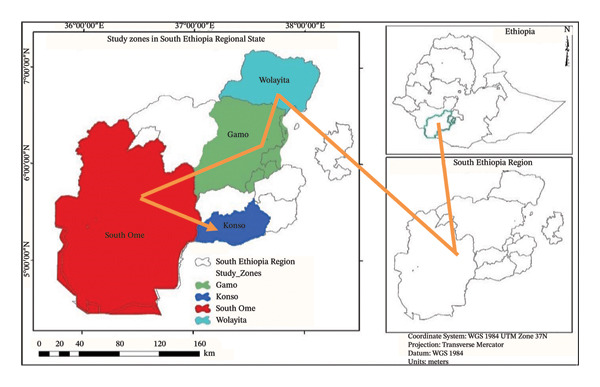
Map of study locations showing Ethiopia, the south region of Ethiopia, and study zones in the south region of Ethiopia. This map was developed using the shapefiles, which were freely available from the website of UNOCHA humanitarian data exchange in the link https://data.humdata.org/dataset/cod-ab-eth [75].

This study was conducted in the hospitals of South Ethiopia [38]. The region had two referral/teaching hospitals, four general hospitals, and 27 primary hospitals [39]. The hospital‐based unmatched case–control study was conducted in South Ethiopia from May 28 to July 27, 2024 (Figure 1).

### 2.2. Eligibility Criteria for Cases and Controls

#### 2.2.1. Cases

A participant who was diagnosed with at least one neonatal adverse event at birth. The adverse fetal birth outcomes listed by the WHO are low birth weight, preterm births, post‐term births, stillbirths, macrosomia, neonatal asphyxia, congenital anomaly, or early neonatal death [2, 40]. The congenital anomaly is further defined as any structural change that is present in infants at birth, which is a physical defect. A low birth weight is defined as a weight of the baby < 2500 g at term birth [41]. A preterm birth is when a fetus is born before 37 completed weeks of gestation [42]. A stillbirth is a fetus with no signs of life after 28 weeks of gestation [6]. Macrosomia is when the weight of the newborn baby is > 4000 g at birth regardless of gestational age [43]. Asphyxia is defined when the 5‐min Apgar score is less than 7 [43]. Early neonatal death: death within 7 days of birth [2]. Mixed adverse fetal birth outcome: if the diagnosis of the fetus was either preterm or low birth weight with asphyxia.

#### 2.2.2. Controls

A participant who was diagnosed with a normal live birth with a gestation of 37 and 40 weeks at birth and a newborn weight between 2500 and 4000 g without signs of asphyxia and/or birth defect.

#### 2.2.3. Exclusion Criteria for Cases and Controls

The women who delivered in the study hospitals but had no laboratory analysis or diagnosis result of birth outcome were excluded. However, critically ill, mentally ill, and women not alive (before the interview) with diagnostic or fetal outcomes were excluded from the study, as they were incapacitated to be interviewed for participant characteristics. Moreover, women who refused consent were excluded.

### 2.3. Sampling Estimation and Procedure

The formula of double population proportion was used to estimate the sample size. As shown in the supporting table 1, using the assumptions of a 95% CI, design effect (2), power (80%), control‐to‐case ratio (2.0), nonresponse rate (20%), expected odds ratio (2.97), and proportion of exposure among cases (35.3%) and controls (15.5%) in Debre Berhan Hospital, North Ethiopia [44]. Hence, the minimum required sample size was 456 women participants who delivered in the study hospitals during the data collection period. The total number of cases was 152, and controls were 304. The design effect (DEFF) = 1 + ICC ^∗^(average cluster size − 1) was the formula used to calculate the design effect. The average cluster size was estimated to be 11 per cluster (daily average number of births in each hospital). Since the ICC was not reported in the former studies, it was estimated to be 0.1 (wider variability or zonal variation) based on a conservative estimation that is commonly assumed between 0 and 0.173 in the study with clinical outcomes [45]. Hence, DEFF = 2.0. For sampling calculation, OpenEpi.com [46] and an additional manual formula were used. The manual formula is given by

(1) n1=Zα/2r+1pq=+Z1−βrp1q1+p2q22rp1−p22,

*n*_2_ = *r*∗n_1_; $\overset{¯}{p}=\left(rP_{2}+P_{1}\right)/\left(r+1\right)$; $\overset{¯}{q}$ = 1−$\overset{¯}{\;p}$; *q*_1_ = 1−*p*_1_; and *q*_2_ = 1−*p*_2_

Parameters,

 
*n* = total sample size, *n*_1_ = sample size in cases, *n*_2_ = sample size in controls.
 
*P*_1_ = proportion of exposure (anemia) among cases (preterm birth), 35.3% [44].
 
*P*_2_ = proportion of exposure (anemia) among controls (not preterm birth) = 15.5% [44].
 
*Z*_1 − β_   = power (the probability that if the two proportions differ, the test will produce a significant difference) (0.84)
 Z_*α*/2_ = standard normal deviate for the two‐sided confidence level (1.96)
 
*r* = control to case ratio (2.0), *n* = 456.

The two‐stage sampling procedure was used in the participant selection. In the first stage, the four public hospitals were selected in four zones, such as the Wolaita Zone, Otona Referral and Teaching Hospital; the Gamo Zone, Arbaminch General Hospital; the Ari Zone, Jinka General Hospital; and the Konso Zone, Karat Primary Hospital. The selection criteria were the existence of admission and inpatient services for obstetrics, Neonatal Intensive Care Unit services, senior obstetricians, senior integrated emergency and obstetrics surgeons, and a load of delivery women (at least 70 births/week on average). Only six hospitals fulfilled the selection criteria in the region, and four were selected randomly.

**FIGURE 2:**
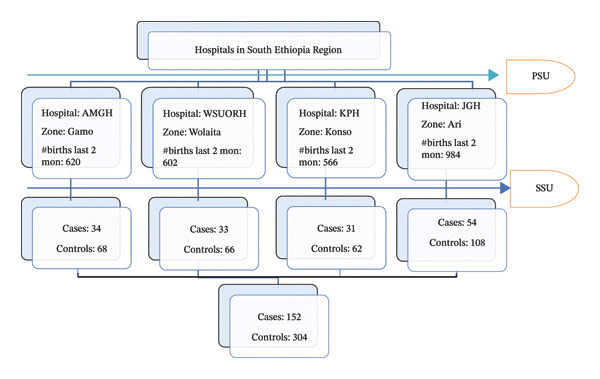
Sampling flow diagram. Note: AMGH: Arba Minch General Hospital; WSUORH: Wolaita Sodo University Otona Referral Hospital; KPH: Konso Primary Hospital; JGH: Jinka General Hospital; Mon: months; #births: number of births; PSU: primary sampling unit; and SSU: secondary sampling unit.

In the second stage, participants were proportionally allocated to each of the four selected hospitals, where the number of births in the previous 2 months before the start of data collection was used to estimate population size. Hospitals’ annual delivery reports were used to estimate 2 months’ population of total delivery. The average number of normal births in the last two months was 2630, and the number of adverse fetal birth outcomes was 164 in the selected hospitals. As the estimated number of women with adverse fetal birth outcomes in the previous 2 months was synonymous with the sample size of the cases. The systematic sampling (the K^th^ value of 1.0, which was K^th^ = N/n, 164/152 = 1.07–1, N = total number of estimated women with adverse fetal birth outcomes in the last 2 months, and n = total number of sampled cases in the last 2 months) was used in the selection of cases sequentially and the selection of controls (the K^th^ value of 8.0, which was K^th^ = N/n, 2630/304 = 8.62–8, N = the total number of estimated normal births, and n = the total number of controls). For the selection of cases and controls, case‐base sampling assumptions were employed. Accordingly, for one case, two controls were selected randomly from a series of visits until a total sample size was achieved in the cases and controls in each of the study hospitals (Figure 2).

### 2.4. Measurement and Data Collection Process

Data was collected using a structured and pretested questionnaire. This questionnaire was partially adapted from a former similar study in the Ethiopia–Somali Region [36]; WHO multicountry studies in low‐ and middle‐income countries [47, 48]; America’s Children and the Environment data tables [49]; Ethiopian DHS 2016 [37]; and other related literature [3, 50].

The data collection process was based on two steps. First, participants screened for eligibility (delivery summary record and client card used). The HGB and adverse fetal birth outcomes data were recorded during eligibility screening. Next, eligible participants were interviewed face‐to‐face for interview questions. The participant’s HGB level and anemia status data were obtained from the request form for hematology laboratory results attached with the client card.

The independent variables include sociodemographic, economic and reproductive variables; anemia status (HGB < 11 g/dL defined as anemic [51], otherwise non‐anemic [52]); and others, such as IFA adherence, IFA intake influencer, deworming received, malaria and intestinal parasitic infection in the current pregnancy, health insurance membership, and insecticide‐treated net (ITN) use in the current pregnancy. Sociodemographic and economic variables include participant age (years), marital status, education and literacy level, occupation, urban‐rural residence status, smoking status, and household income status. Reproductive variables include menstrual status, parity, gravidity, inter‐pregnancy intervals, mode of delivery, adherence to IFA, and history of former adverse fetal birth outcomes. The SAMANTA tool was used to measure the history of heavy menses. A participant had heavy menses if she scored 3 or more, such as menstrual bleeding for > 7 days per month (3 points); ≥ 3 heavy menses days as she felt (1 point); menses abundance bothered her (3 points); spotting clothes at night (1 point); staining the chair or sofa (1 point); and increased frequency of changing pads (1 point). A participant was considered to have better adherence to IFA if they received at least 4 IFA supplements every week during the last month of IFA supplementation in pregnancy [53]; otherwise, they were classified as non‐adherent, as suggested elsewhere [54]. The outcome variable was a composite index of preterm birth, low birth weight, stillbirth, macrosomia, asphyxia, or congenital anomalies. An outcome variable with any one of the “yes” responses was indexed as “had adverse birth event”; otherwise, “had no adverse birth event” (supporting table‐2).

The data collection was face‐to‐face. The KoboToolbox application was installed on the mobile tablets of data collectors after 3 days of intensive training. The application uses KoboToolbox to collect high‐quality data electronically using mobile tablets [55]. The data collection process was supervised by two skilled personnel who had an MSc degree in nursing with a maternity nursing specialty. Eight enumerators who had a BSc in midwifery were temporarily recruited as data collectors for 2 months.

### 2.5. Data Quality Control

To ensure data quality, training was given to supervisors and enumerators. Training was given on study methods, data collection tools (word by word), and how to use the KoboToolbox application. A mock interview was performed between trainees and each other. A pretest was employed on 12 cases and 24 control women at birth in an unselected hospital in the Gamo Zone (such as Dil Fana Hospital), with a case–control ratio of 1:2. Every day, completed data were received by the Kobo server, and feedback was offered by central personnel before next‐day data collection started. The report on birth outcome and anemia was based on the diagnosis performed by senior gynecologists in the study hospitals. Major local languages (Amharic, Gamootho, Konsigna, and Wolaitato) were used during the data collection process and back‐translated to English during the analysis period.

### 2.6. Analysis

Data on the Kobo server was exported into Excel and imported into SPSS v25 for cleaning and statistical analysis. The descriptive analysis was performed for independent and dependent variables and presented in tables and figures. A composite index was used to produce a single outcome variable for adverse fetal birth outcomes, as did elsewhere [15, 17, 31–33, 36, 37, 56–60]. Since this study has a binary outcome, an unadjusted logistic regression model was fitted between an outcome and each of the independent variables, using a *p* value < 0.25 to justify unadjusted associations. To control confounders, the adjusted logistic regression model was the final model fitted to test the statistical associations between the outcome variable and anemia status of the participants by adjusting for BMI, malaria infection, education status, IFA adherence, age of the woman, and history of heavy menses and intermenstrual bleeding. The *p* value < 0.05 was used to confirm the final statistical associations. The multicollinearity effect was checked using the variance inflation factor in SPSS. Questionnaire validation was implemented during the pretest period. Model adequacy and multiple testing errors were checked using the Hosmer and Lemeshow goodness‐of‐fit test in SPSS (*p*‐value: 0.31) and model summary statistics (−2 log‐likelihood: 341.15). There was no evidence of a substantial clustering effect between hospitals (ICC, 1.54e − 33; std error, 7.13e − 18). Since the missing was completely at random, the missing data was handled based on a complete‐case analysis, as recommended elsewhere [61]. Final analysis was performed based on the remaining data set. Accordingly, 5% of the data from the control group was missing from the final analysis due to consent denial.

## 3. Results

### 3.1. Sociodemographic Characteristics

A total of 433 women at birth participated in the survey, of which 152 were cases and 281 were controls. The 5% missed participants from the controls could be related to rushing to go home seeking cultural services, provided that delivery was normal, as reported in Ethiopia [62]. The mean age of participants with standard deviation among cases was 27.9 ± 5.4, and controls was 26.7 ± 6.0. The majority of women were married among cases (145, 95.4%) and controls (271, 96.4%). About six out of 10 participants (*n* = 97, 63.8%) among cases attended school until the elementary level, and nearly five out of ten (*n* = 129, 45.9%) attended among controls. The farmer occupation was the dominant among the cases (*n* = 116, 76.3%), and the housewife occupation was the dominant among the controls (*n* = 175, 62.3%). The participant’s median daily income was 1.76 USD ± 2.45 SD among the cases and 1.76 USD ± 2.23 SD among the controls. More than half of participants in the cases (*n* = 82, 53.9%) and the controls (*n* = 183, 65.1%) were not members of community‐based health insurance (Table 1).

**TABLE 1:** Sociodemographic characteristics of women between cases and controls in public health institutions of the South Ethiopia Region (*n* = 433), May 28 to July 27, 2024.

Variables and category	Cases, *n* = 152 (%)	Controls, *n* = 281 (%)	*p* value
Age group			< 0.05
< 20 years	3 (2.0)	29 (10.3)	
20–35 years	135 (88.8)	230 (81.9)	
> 35 years	14 (9.2)	22 (7.8)	
Marital status			> 0.25
Married	145 (95.4)	271 (96.4)	
Others^∗^	7 (4.6)	10 (3.6)	
Level of education			< 0.05
Primary or no formal school	97 (63.8)	129 (45.9)	
High school	34 (22.4)	77 (27.4)	
More than high school	21 (13.8)	75 (26.7)	
Level of literacy			< 0.05
Secondary school completed and higher	31 (20.4)	97 (34.5)	
Below primary and can read a whole sentence	26 (17.1)	41 (14.6)	
Below primary and can read part of sentences	50 (32.9)	66 (23.5)	
Below primary and cannot read at all	33 (21.7)	66 (23.5)	
No card with required language	12 (7.9)	11 (3.9)	
Occupation			< 0.05
Housewife	55 (36.2)	105 (37.4)	
Farmer	61 (40.1)	70 (24.8)	
Government employed	12 (7.9)	46 (16.4)	
Merchant	13 (8.6)	19 (6.8)	
Others^#^	11 (7.2)	41 (14.6)	
Place of residence			< 0.05
Urban	59 (38.8)	172 (61.2)	
Rural	93 (61.2)	109 (38.8)	
Daily household income in USD			> 0.25
< 1.25	52 (34.2)	84 (29.9)	
1.25–2.5	58 (38.2)	89 (31.7)	
2.5–4	25 (16.4)	62 (22.1)	
4–10	15 (9.9)	41 (14.6)	
> 10	2 (1.3)	5 (1.8)	
Health insurance membership status			< 0.05
Insured	70 (46.1)	98 (34.9)	
Non‐insurance	82 (53.9)	183 (65.1)	

*Note:* others^#^: private employees, students, and private unions; *p* values are using chi‐square statistics for unadjusted associations.

Abbreviation: USD, United States dollar.

^∗^Separated, widowed, and divorced.

### 3.2. Obstetrics, Behavioral, and Clinical Characteristics of Participants

Approximately four out of 10 women among cases (*n* = 63, 41.4%) and slightly more than one out of five among controls (*n* = 65, 23.1%) had a history of heavy menses in the last year before the recent pregnancy, and a history of malaria infection was higher among cases (*n* = 60, 39.5%). The majority of participants had no history of intermenstrual bleeding among cases, 85 (55.9%), and controls, 213 (75.8%), in the last year before the recent pregnancy. About one out of five participants in cases (*n* = 33, 21.7%) and seven out of 20 in controls (*n* = 96, 34.2%) were primiparous. About four out of five participants with adverse fetal birth outcomes (*n* = 121, 79.6%) and 13 out of 20 controls (*n* = 187, 66.5%) were multigravida. Approximately two‐thirds of women among cases (*n* = 96, 63.2%) and four‐fifths among controls (*n* = 218, 77.6%) had attained birth through spontaneous vaginal delivery. About half of participants (*n* = 69, 51.9%) among cases and nearly one out of 10 participants among controls (*n* = 38, 16.2%) had a history of adverse fetal birth outcomes in any one of their former births. Nearly negligible study participants were using tobacco (*n* = 3, 2%) among cases and (*n* = 2, 0.7%) among controls. About eight out of 10 women among cases (*n* = 129, 84.9%) and (*n* = 243, 86.5%) among controls had reported not receiving deworming in their recent pregnancy (Table 2).

**TABLE 2:** Obstetrics and related characteristics of participants in public health institutions of the South Ethiopia Region (*n* = 433), May 28 to July 27, 2024.

Variables	Category	Cases, *n* = 152 (%)	Controls, *n* = 281 (%)	*p* value
History of heavy menstrual bleeding (last 1 year before the current pregnancy)	Yes	63 (41.4)	65 (23.1)	< 0.001
	No	89 (58.6)	216 (76.9)	

History of intermenstrual bleeding (last 1 year before the current pregnancy)	Yes	67 (44.1)	68 (24.2)	< 0.001
	No	85 (55.9)	213 (75.8)	

Parity	1	33 (21.7)	96 (34.2)	0.018
	2–3	69 (45.4)	116 (41.3)	
	4+	50 (32.9)	69 (24.6)	

Gravidity	1	31 (20.4)	94 (33.5)	0.011
	2–3	68 (44.7)	115 (40.9)	
	4+	53 (34.9)	72 (25.6)	

Inter‐birth interval	< 24 months	56 (47.1)	71 (38.2)	0.16
	24–36 months	27 (22.7)	60 (32.3)	
	> 36 months	36 (30.3)	55 (29.6)	

Mode of delivery in the current birth	SVD	96 (63.2)	218 (77.6)	0.001
	CS or other operation^∗^	56 (36.8)	63 (22.4)	

Adverse event in any one of former births	Yes	69 (45.4)	38 (13.5)	< 0.001
	No adverse event	64 (42.1)	196 (69.8)	
	No former birth	19 (12.5)	47 (16.7)	

Influence on IFA intake	Self	42 (27.6)	71 (25.3)	0.59
	HCWs, peers and family	110 (72.4)	210 (74.7)	

Deworming in the current pregnancy	Received	23 (15.1)	38 (13.5)	0.65
	Not received	129 (84.9)	243 (86.5)	

History of intestinal parasites in the current birth	Yes	44 (28.9)	57 (20.3)	0.042
	No	108 (71.1)	224 (79.7)	

History of malaria in the current pregnancy	Yes	60 (39.5)	48 (17.1)	< 0.001
	No	92 (60.5)	233 (82.9)	

Use ITN in the current pregnancy	Yes	103 (67.8)	188 (66.9)	0.86
	No	49 (32.2)	93 (33.1)	

Community view towards IFA supplementation in pregnancy	Positive	120 (78.9)	237 (84.3)	0.16
	Negative	32 (21.1)	44 (15.7)	

Smoking cigarette/tobacco	Yes	3 (2.0)	2 (0.7)	0.24
	No	149 (98.0)	279 (99.3)	

Abbreviations: CS: cesarean section; HCW: healthcare worker; IFA: iron–folic acid; ITN: insecticide‐treated net; SVD: spontaneous vaginal delivery.

### 3.3. Anemia and Adverse Events at Birth

**FIGURE 3:**
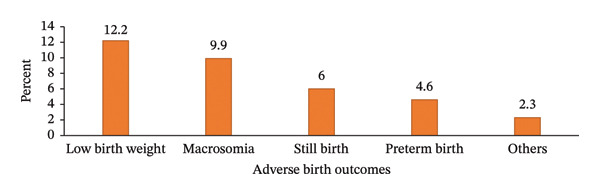
Types of adverse fetal birth outcomes among cases in four study hospitals of South Ethiopia Region (cases = 152), May 28 to July 27, 2024. Note: Percentage indicates percent of each type of adverse birth events out of the total number of participants; Others: asphyxia and/or anomaly and/or post‐term birth.

The proportion of adverse fetal birth outcomes women faced ranges from 10 (2.3%) others (asphyxia or anomalies or post‐term or mixed (such as preterm birth/low birth weight with asphyxia) fetal birth outcomes) to 53 (12.2%) low birth weight (Figure 3). As shown in supporting table 3, the types of adverse events were shown by the anemia status that women faced immediately before birth. Based on unadjusted statistical associations, the proportion of anemia status (immediately before birth) was significantly higher in the cases than in the controls (68 (44.7%) versus 48 (17.1%), *p* < 0.001). More specifically, the proportion of anemia was higher in women with stillbirths (*p* < 0.001, unadjusted) and macrosomia (*p* = 0.002, unadjusted). However, evidence did not suggest a difference in the proportion of anemia in women with low birth weight (*P* = 0.11, unadjusted) and preterm births (*p* = 0.48, unadjusted). Participants who faced stillbirth with a history of anemia were 19 (73.1%) with a 95% CI of 56%–90.1% (Figures 3 and supporting table 3).

### 3.4. Factors Associated With Adverse Fetal Birth Outcomes

The study result showed an association between anemia and adverse fetal birth outcomes. Women with anemia immediately before birth had 2.1 (AOR: 95% CI: 1.21, 3.61) higher odds of adverse fetal birth outcomes than women without anemia. Regarding IFA adherence during the current pregnancy, women who had a better history of adherence had 53% (AOR: 0.47; 95% CI: 0.28, 0.79) lower odds of adverse fetal birth outcomes than those with poor adherence.

The adverse fetal outcome had an association with the education status of women. The low‐educated women had 2.67 (AOR: 95% CI: 1.32, 5.38) higher odds of adverse fetal birth outcomes than the highly educated (college or higher). Having a history of adverse fetal birth outcomes in the former birth impacts the occurrence of adverse fetal birth outcomes in the current birth. Women having a history of adverse fetal birth outcomes had 3.62 (AOR: 95% CI: 2.09, 6.28) higher odds of adverse fetal birth outcomes in the current birth than those having no history. Moreover, women having a history of intermenstrual bleeding in the last year before the current pregnancy had 2.7 (AOR: 95% CI: 1.24, 5.86) higher odds of adverse fetal birth outcomes than those having no history (Table 3).

**TABLE 3:** Multivariable binary logistic regression model to show statistical associations of adverse fetal birth outcomes and explanatory variables among women at birth in public health institutions of the South Ethiopia Region (*n* = 433), May 28 to July 27, 2024.

Variable	Cases, *n* = 152 (%)	Controls, *n* = 281 (%)	COR (95% CI)	AOR (95% CI)	*p* value
Anemia status^#^					< 0.01
Anemia	68 (44.7)	48 (17.1)	3.93 (2.52, 6.14)^∗^	**2.1 (1.21, 3.61)** ^∗∗^	
No anemia	84 (55.3)	233 (82.9)	1	1	
Adherence with IFA					< 0.01
Good	62 (40.8)	156 (55.5)	0.55 (0.37, 0.82)^∗^	**0.47 (0.28, 0.79)** ^∗∗^	
Poor	90 (59.2)	125 (44.5)	1	1	
Education status of woman					< 0.01
Elementary	97 (63.8)	129 (45.9)	2.68 (1.55, 4.66)^∗^	**2.67 (1.32, 5.38)** ^∗∗^	
High school	34 (22.4)	77 (27.4)	1.58 (0.84, 2.96)^∗^	1.8 (0.87, 3.71)	
More than high school	21 (13.8)	75 (26.7)	1	1	
Adverse birth event in the former births					< 0.001
Yes	69 (51.9)	38 (16.2)	5.32 (3.33, 8.49)^∗^	**3.62 (2.09, 6.28)** ^∗∗^	
No+	83 (48.1)	243 (83.8)	1	1	
History of heavy menses					> 0.05
Yes	63 (41.4)	65 (23.1)	2.35 (1.54, 3.60)^∗^	1.62 (0.72, 3.64)	
No	89 (58.6)	216 (76.9)	1	1	
History of malaria infection (current pregnancy)					> 0.05
Yes	60 (39.5)	48 (17.1)	3.17 (2.02, 4.96)^∗^	1.49 (0.86, 2.59)	
No	92 (60.5)	233 (82.9)	1	1	
History of intermenstrual bleeding					< 0.05
Yes	67 (44.1)	68 (24.2)	2.47 (1.62, 3.76)^∗^	**2.7 (1.24, 5.86)** ^∗∗^	
No	85 (55.9)	213 (75.8)	1	1	
Age of the woman at birth					> 0.05
Less than 20 years	3 (2.0)	29 (10.3)	0.16 (0.04, 0.64)^∗^	0.41 (0.08, 1.94)	
20–35 years	135 (88.8)	230 (81.9)	0.92 (0.46, 1.86)	2.25 (0.95, 5.32)	
Greater than 35 years	14 (9.2)	22 (7.8)	1	1	
History of intestinal parasites (current pregnancy)					> 0.05
Yes	44 (28.9)	57 (20.3)	1.6 (1.01, 2.52)^∗^	0.93 (0.51, 1.7)	
No	108 (71.1)	224 (79.7)	1	1	

*Note:* No+: no history of adverse birth events in the former births or no former births. Anemia status^#^: adjusted for adherences, education, former adverse events, heavy menses, malaria, intermenstrual bleeding, age, and intestinal parasites.

Abbreviations: AOR: adjusted odds ratio; CS: cesarean section; COR: crude odds ratio; IFA: iron–folic acid; SVD: spontaneous vaginal delivery.

^∗^Significant by unadjusted logistic regression (*p* < 0.25).

^∗∗^Statistically significant associations (*p* < 0.05), (*p* < 0.01), and (*p* < 0.001).

## 4. Discussions

This study determined the associations of adverse fetal birth outcomes among women who delivered at public health institutions in the southern Ethiopian Region. The most common adverse fetal birth outcomes observed were low birth weight, macrosomia, stillbirths, and preterm birth. Anemia in pregnant women; IFA adherence; previous history of adverse fetal birth outcomes; history of intermenstrual bleeding; and educational level of women were characteristics that predicted adverse fetal birth outcomes.

In this study, the anemia status of women was one of the important associated factors with adverse fetal birth outcomes. The current study finding was in line with a prospective cohort study in Tanzania (stillbirth as an outcome) [63], a population‐based survey in Bangladesh [25], and Ethiopian study reports [35, 36]. In the Ethiopian–Somalia Region, higher adverse fetal birth outcomes were reported, where the prevalence of anemia was higher in this region than in others throughout Ethiopia [29, 30, 57]. When a woman becomes anemic, her blood’s oxygen‐carrying capacity deteriorates, which can cause damage to the fetus; insufficient oxygen supply may lead to termination of gestation, resulting in preterm birth, low birth weight, or fetal demise [3, 64].

This study observed that women who had better adherence to the IFA regimen had shown a protective association with adverse fetal birth outcomes. Even though the evidence was unclear to justify the effect of adherence to IFA during pregnancy on adverse fetal birth outcomes in Ethiopia so far [15, 17, 31–33, 36, 37, 56–60], the current study justified the existence of statistical associations. Poor adherence to IFA could be one of the contributors to the incidence of anemia in pregnancy, as reported by combined DHS evidence of India and Cambodia [65]. Anemia due to non‐adherence to IFA could have contributed to the occurrence of adverse fetal birth outcomes [21, 25, 31, 63, 66]. Evidence supports that regions with low maternal adherence to IFA intake during pregnancy reported high anemia prevalence in pregnancy, such as 2.3% versus 56.8% in Ethiopia’s Somali Regional Administration [37], 1.4% versus 44.2% in Burundi, 7.8% versus 40.3% in Kenya, and 5% versus 46.5% in the Democratic Republic of Congo [52, 67].

In the current study, women’s better educational attainment had a protective association with adverse fetal birth outcomes. The demographic and health survey from 10 sub‐Saharan African countries similarly justified that the risk factors for adverse fetal birth outcomes were low levels of maternal education and illiteracy [11]. The U.S. Children and Environment Protection Agency reported that some maternal characteristics, including educational status, had a positive association with low birth weight and preterm birth outcomes [40]. It was in agreement with the condition that women with better education attainment were healthier than their counterparts [68]. It was suggested that better education status had a linear association with maternal services and related outcomes [69].

As revealed in this study, women who had adverse fetal birth outcomes in any one of the former births were likely to be repeated in the current birth. The association between a previous history of adverse fetal birth outcomes and adverse fetal birth outcomes in the current birth was not evaluated in the former Ethiopian research reports [15, 17, 36, 56]. The result was consistent with the Tanzanian prospective study, where women with a history of stillbirth were likely to have it reoccur [63], and no association was observed in the Ethiopian cross‐sectional study finding in Hawassa [60]. The recurrence of adverse fetal birth outcomes could be related to biological [70], genetic [71], anatomical, lifestyle [71, 72], or placental factors [70–72].

In the current study, women with a history of intermenstrual bleeding were more likely to have a composite outcome of adverse fetal birth outcomes. Consistently, in an Australian survey, women who experienced stillbirth and preterm labor reported higher rates of irregular menstrual cycles and intermenstrual bleeding [73]. This could be related to the existing knowledge and evidence that bleeding is one of the causes of anemia, among others [64]. This maternal anemia could possibly have contributed to the occurrence of adverse fetal birth outcomes [21, 63, 66]. This was because maternal anemia could result in fetal anemia (too few RBCs) or polycythemia (too many immature RBCs), which could possibly result in a low‐birth‐weight baby [40, 64].

### 4.1. Public Health Implications

The existing data suggest that women with anemia immediately before birth had an association with overall adverse fetal birth outcomes. Based on the given evidence, anemia prevention interventions should be considered for women during the antenatal visit. For the successful improvement of anemia prevention strategy in pregnancy, the pregnant women may be counseled to adhere to the IFA regimen, and risk (formerly adverse fetal outcome) screening activities in the antenatal period could be encouraged. The antenatal women’s HGB status should be evaluated frequently to ensure the status of anemia and adherence to IFA during the late antenatal visit.

### 4.2. Strengths and Limitations of the Study

#### 4.2.1. Strengths

Using the hospital client card to access participant adverse fetal birth outcomes and anemia data was useful to minimize recall bias. Being institution‐based could have helped in getting relatively accurate data in the hospitals equipped with improved facilities and diagnostic instruments. Moreover, the study included nighttime births, as data collectors were there throughout the day and night in the study hospitals.

#### 4.2.2. Limitations

Using participant medical records (for anemia and adverse events) could have caused missed or misreported data from the physician’s report, and reliance on secondary data might have affected the results. Recall bias from oral reports of variables (suppose adherences) was possible, though minimized by asking recent history. Even though obtaining interview data was not possible, the exclusion of incapacitated participants (women not alive, critically ill, or mentally ill) and those who refused consent could have affected the result. Temporal limitations might have affected the potential to definitively relate the outcome to the exposure status. The association could be affected by a one‐time HGB measurement, as this could not show women’s previous progressive status of anemia in the current pregnancy and related pregnancy outcomes.

## 5. Conclusions

Anemia has an association with adverse fetal birth outcomes. Adherence to IFA supplementation had a protective association with adverse fetal birth outcomes. Pregnant women should be counseled on anemia prevention strategies in pregnancy, such as the utilization of iron‐rich diets and monitoring and counseling to adhere to the IFA regimen in the antenatal follow‐up. Future cohort studies are recommended to establish causal associations and assess the timing and severity of anemia on distinct birth outcomes.

**Nomenclature**
ANC: Antenatal care
BMI: Body mass index
DHS: Demographic and Health Survey
HGB: Hemoglobin
IFA: Iron–folic acid
ITN: Insecticide‐treated net
LMIC: Low‐ and middle‐income countries
RBC: Red blood cell
USA: United States of America
USD: United States dollar
U.S. EPA: United States Environmental Protection Agency
WHO: World Health Organization

## Author Contributions

The contributions of the authors were substantial in the process and development of the manuscript. Serawit Lakew Chillo: developed the concept; reviewed the literature; designed the study, protocol development, and monitoring; performed data analysis, report writing, sequencing, and manuscript drafting. Endrias Markos Woldesemayat: searched the literature and participated in data analysis, report writing, and manuscript drafting. Mesay Hailu Dangisso: reviewed the literature and participated in designing, report writing, sequencing, and drafting the manuscript.

## Funding

This research project was funded by Arba Minch University and Hawassa University. The funding was used for data collection.

## Disclosure

All the authors approved the final manuscript.

## Ethics Statement

Declarations under the principles of Helsinki and the Ethiopian National Research Ethics Guidelines were maintained in the process [74]. An ethical approval letter was received from the institutional review board (IRB) of Arba Minch University, registration number IRB/23124/2024, dated January 17, 2024, and protocol number SL23124. A written permission letter was secured from district healthcare administration offices for each of the study health facilities. Written informed consent was obtained from all the participants, including emancipated minors (based on the national guideline [74]). Data collectors informed the women of their right to refuse or terminate participation at any time they wished during the interview process without a precondition. Moreover, the privacy and confidentiality of participants were maintained throughout the process of data collection and management.

## Consent

The authors have nothing to report.

## Conflicts of Interest

The authors declare no conflicts of interest.

## Supporting Information

Additional supporting information can be found online in the Supporting Information section.

## Supporting information


**Supporting Information 1** Supporting Table 1: Summary of minimum sample size required for determinants of adverse fetal birth outcomes using exposure variables and the assumptions considered.

**Supporting Information 2** Supporting Table 2: Description and measurement of study variables in the study of risk of adverse fetal birth outcomes in the Southern Regional State of Ethiopia.

**Supporting Information 3** Supporting Table 3: Percent distribution of adverse fetal birth outcomes by anemia status of women delivering at hospitals of the South Ethiopia Regional State (*n* = 433), May 28 to July 27, 2024.

## Data Availability

The datasets used and/or analyzed during the current study are available from the corresponding author upon reasonable request.
