# Supplementary material for: Association of Maternal Anemia and Adverse Fetal Birth Outcomes Among Women Who Gave Birth at Public Hospitals in Southern Ethiopia: An Unmatched Case–Control Study
Source: Anemia. 2026 Jul 20;2026:9108578. doi: 10.1155/anem/9108578 (PMC13385512; doi:10.1155/anem/9108578)
Supplement: Supplementary file 1 — Supporting Information 1 Supporting Table 1: Summary of minimum sample size required for determinants of adverse fetal birth outcomes using exposure variables and the assumptions considered. [file ANEM-2026-9108578-s002.docx]

Supplementary table-1: Summary of minimum sample size required in determinants of adverse birth outcomes using exposure variables and the assumptions considered.

| Exposure variables | | Outcome | | Level of significance;  Design effect;  Formula;  Non-response rate; Control to case ratio | Sample size |
| --- | --- | --- | --- | --- | --- |
|  |  | Adverse birth outcome | |  |  |
|  |  | Yes | No |  |  |
| Age (years) [97] | ≥ 35 years | 22 (P1=38.6%) | 9  (P2=3.5%) | 95%; 2.0; double population proportion; 20%; 2.0 | 127 |
|  | < 35 years | 35 | 247 |  |  |
| Residence [97] | Rural  (exposed) | 40 (P1=70.2%) | 59  (P2=23%) | 95%; 2.0; double population proportion; 20%; 2.0 | 108 |
|  | Urban | 17 | 197 |  |  |
| Fever (malaria) [98] | Yes (exposed) | 43 (P1=32.6%) | 34 (P2=10.5%) | 95%; 2.0; double population proportion; 20%; 2.0 | 322 |
|  | No | 89 | 289 |  |  |
| High risk pregnancy [99] | Yes (exposed) | 36 (P1=57.1%) | 57  (P2=8.6%) | 95%; 2.0; double population proportion; 20%; 2.0 | 92 |
|  | No | 27 | 605 |  |  |
| History of stillbirth [99] | Yes (exposed) | 29 (P1=46%) | 73  (P2=11%) | 95%; 2.0; double population proportion; 20%; 2.0 | 159 |
|  | No | 34 | 589 |  |  |
| Type of delivery [99] | Non-SVD (exposed) | 49 (P1=77.8%) | 145 (P2=21.9%) | 95%; 2.0; double population proportion; 20%; 2.0 | 79 |
|  | SVD | 14 | 517 |  |  |
| Anemia [99] | Yes (exposed) | 18 (P1=35.3%) | 41 (P2=15.5%) | 95%; 2.0; double population proportion; 20%; 2.0 | **456** |
