# Supplementary material for: Association of Maternal Anemia and Adverse Fetal Birth Outcomes Among Women Who Gave Birth at Public Hospitals in Southern Ethiopia: An Unmatched Case–Control Study
Source: Anemia. 2026 Jul 20;2026:9108578. doi: 10.1155/anem/9108578 (PMC13385512; doi:10.1155/anem/9108578)
Supplement: Supplementary file 2 — Supporting Information 2 Supporting Table 2: Description and measurement of study variables in the study of risk of adverse fetal birth outcomes in the Southern Regional State of Ethiopia. [file ANEM-2026-9108578-s003.docx]

Supplementary_table_2: Description and measurement of study variables in the study of risk of adverse birth outcomes in the Southern Regional State of Ethiopia.

| **Variables** | **Descriptions** | | | **Measurements** |
| --- | --- | --- | --- | --- |
| **Outcome variables** | | | | |
| Adverse birth outcome | | Composite indices will be used. Anyone with ‘yes’ response will be ‘having adverse events’, otherwise ‘no adverse event’ | | ‘Adverse event’ … (1),  ‘No adverse event’ … (0) |
| Congenial anomaly | | Any detectable structural defect at birth | | ‘Yes’… (1), ‘No’ … (0) |
| Low birth weight | | Weight of the baby < 2500 grams at birth | | ‘Yes’… (1), ‘No’ … (0) |
| Preterm birth | | Fetus born before 37 completed weeks of gestation | | ‘Yes’… (1), ‘No’ … (0) |
| Still birth | | Fetus born with no signs of life after 28 weeks of gestation | | ‘Yes’… (1), ‘No’ … (0) |
| Macrosomia | | Weight of the newborn baby > 4000 grams at birth regardless of gestational age | | ‘Yes’… (1), ‘No’ … (0) |
| Asphyxia | | A 5-minute Apgar score < 7 out of 10 | | ‘Yes’… (1), ‘No’ … (0) |
| **Independent variables** | | | | |
| Age | Age of woman in completed years as reported orally | | | Age in completed years |
| Parity | Women total number of births ever | | | Number recorded |
| Gravidity | Women total number of pregnancies ever | | | Number recorded |
| Birth interval | Inter-birth interval between the current and last birth | | | Number recorded |
| Menstrual status | History of heavy menstrual bleeding | | | ‘Yes’… (1), ‘No’ … (0) |
|  | History of inter-menstrual bleeding | | | ‘Yes’… (1), ‘No’ … (0) |
| BMI | Measured as the ratio of weight (kg) to height (m^2^) | | | Recorded in kg/m^2^ |
| Occupation | Major occupation of women | | | One major occupation selected  (1)Housewife, (2)Farmer,  (3)Government employee (4) Merchant (5) Others, specify_ |
| Education status | Women highest level of school achievement | | | Recorded in completed years |
| Level of literacy | Six literacy level recorded | | Coded as (1) Higher than secondary education,  (2) No schooling, primary or secondary schooling and Can read a whole sentence  (3) >> Can read part of a sentence (4) >> Cannot read at all, (5) >> No card with required language, (6) >> Blind or visually impaired | |
| Marital status | Current marital status of woman. If married but live separately record as married. If divorced but not legally, recorded as married. | | | Coded as (1) Married, (2) Separated, (3) Divorced, and (4) Widowed, (5) Single |
| Place of residence | Place of participants permanent residence | | | Coded as (1) urban, (2) rural |
| Income | Daily income by household level | | | Amount in US Dollar |
| Insurance | Membership of household/family in community-based health insurance coverage | | | Coded as (1) Yes, (2) No |
| Adherence | If woman received at least 4 tablets in every week in the last 1 month of supplementation in the last pregnancy was considered as adhered. | | | 1. Adhered 2. Not adhered |
| Smoking | Current smoking status of women | | | ‘Yes’… (1), ‘No’ … (0) |
| Mode of delivery | Mode of delivery in the current birth | | | (1)SVD, (2) CS (3) others |
| History of adverse event | History of adverse birth events in any one of the former births | | | ‘Yes’… (1), ‘No’ … (0) |
| IFA influence | Influence on IFA intake  (multiple response) | | | (1)Self (2)family (3)peer (4)neighbor (5) HCWs (6)others |
| Deworming | Women received deworming in the recent pregnancy | | | ‘Yes’… (1), ‘No’ … (0) |
| Intestinal parasite infection | History of intestinal parasite infection in the current birth | | | ‘Yes’… (1), ‘No’ … (0) |
| Malaria infection | History of malaria infection in the recent pregnancy | | | ‘Yes’… (1), ‘No’ … (0) |
| ITN use | ITN consistently used in the recent pregnancy | | | ‘Yes’… (1), ‘No’ … (0) |
| Community view on IFA | Community elders view towards IFA supplementation in pregnancy | | | (1) Positively viewed  (2) Negatively viewed |
