# Supplementary material for: Association of Maternal Anemia and Adverse Fetal Birth Outcomes Among Women Who Gave Birth at Public Hospitals in Southern Ethiopia: An Unmatched Case–Control Study
Source: Anemia. 2026 Jul 20;2026:9108578. doi: 10.1155/anem/9108578 (PMC13385512; doi:10.1155/anem/9108578)
Supplement: Supplementary file 3 — Supporting Information 3 Supporting Table 3: Percent distribution of adverse fetal birth outcomes by anemia status of women delivering at hospitals of the South Ethiopia Regional State (n = 433), May 28 to July 27, 2024. [file ANEM-2026-9108578-s001.docx]

Supplementary Table 3: Percent distribution of adverse birth events by anemia status of women delivering at hospitals of south Ethiopia Regional State (n = 433), May 28 to July 27, 2024.

| **Participant anemia Status** | **Types of adverse birth events** | | | | | | | | | |
| --- | --- | --- | --- | --- | --- | --- | --- | --- | --- | --- |
|  | **Stillbirths** | | **LBW** | | **PTB** | | **Macrosomia** | | **Others** | |
|  | Yes | No | Yes | No | Yes | No | Yes | No | Yes | No |
| Anemic | 19  (73.1) | 97  (23.8) | 19  (35.8) | 97 (25.5) | 4  (20) | 112  (27.1) | 20  (46.5) | 96  (24.6) | 6  (60) | 110  (26) |
| Non anemic | 7  (26.9) | 310  (76.2) | 34  (64.2) | 283  (74.5) | 16  (80) | 301  (72.9) | 23  (53.5) | 294  (75.4) | 4  (40) | 313  (74) |
